# Expanding and Retracting From the Self: Gains and Costs in Switching Self-Associations

**DOI:** 10.1037/xhp0000125

**Published:** 2015-09-07

**Authors:** Haixu Wang, Glyn Humphreys, Jie Sui

**Affiliations:** 1 Department of Psychology, Tsinghua University; 2 Department of Experimental Psychology, Oxford University; 3 Department of Psychology, Tsinghua University, and Department of Experimental Psychology, Oxford University

**Keywords:** self-association, switching, memorial glue

## Abstract

We report 2 experiments to assess the strength of forming and breaking associations to the self, familiar others, and unfamiliar others in a simple shape–label matching task. In each experiment, participants first formed shape–person associations (e.g., triangle-self). Subsequently, they had to relearn the associations with the shapes and labels rearranged (self→stranger in Experiment 1; self→friend in Experiment 2) and they carried out a matching task in which they judged whether shape–label stimuli were as newly instructed or re-paired. There were faster responses and fewer errors on match trials for newly formed self-associated stimuli. In contrast, after switching, reaction times were slower and accuracy was reduced on mismatch trials involving shapes previously associated with the self. The strength of the self-advantage in forming the new association on match trials correlated with the difficulty in switching from the old self-associated shape on mismatch trials. The results indicate that self-reference enhances the binding of associations in memory; this facilitates associations to new stimuli, but there is a cost of interference from old associations.

Self-biases are pervasive, affecting memory (Cunningham, Turk, Macdonald, & Macrae, 2008; Rogers, Kuiper, & Kirker, 1977), attention (Keyes & Brady, 2010; Tong & Nakayama, 1999), and perceptual matching (Sui, He, & Humphreys, 2012). Self-biases typically take the form of processing enhancements. For example, memory is better to items encoded in relation to one’s self compared with items encoded in relation to others (Rogers et al., 1977). In face perception, responses to one’s own face are faster and more accurate than responses to the face of a familiar other (Ma & Han, 2010). However, self-biases can also lead to processing costs, for example, when self-related information must be ignored. Brédart, Delchambre, and Laureys (2006) reported that the presence of the participant’s own face and name can automatically attract attention away from task-related targets and can hurt performance.

One way of conceptualizing the benefits and costs to self-associated stimuli in a task is to suggest that self-association is akin to having greater “memorial glue.” When associations are formed in memory, self-reference enhances the binding process so that different elements are more strongly linked and retrieved together, and this enhances memory performance (Rogers et al., 1977). For example, Cunningham, Brady-Van den Bos, and Turk (2011) asked participants to categorize items as belonging to themselves or to someone else based on the color of the stimulus (e.g., red cues meant self-owned objects, and blue cues referred to objects owned by others). After encoding the items in relation to ownership by the self versus others, participants carried out a surprise memory task. Cunningham et al. found that participants had better memory for self-owned items compared with other-owned items irrespective of whether self-owned items were owned in reality or not. Moreover, for remembered items, participants were more accurate at judging that the stimuli had been categorized in relation to the self than at judging whether the stimuli had been characterized in relation to another person. This enhancement of source memory is consistent with the better binding of elements to their context. Sui and Humphreys (2013) further reported self-reference enhanced source memory even in an amnesic individual who showed no effects of semantic elaboration on memory. This suggests that self-reference effects are independent of semantic elaboration.

In this study, we tested two ideas that stem from the proposal that self-reference increases binding in memory. The tests were based on conditions in which participants form new associations between geometric shapes and labels referring to the self, a familiar other, or an unfamiliar other (e.g., Sui et al., 2012). For example, Sui et al. (2012) had participants learn associations between simple shapes (square, triangle, circle) and personal labels (stranger, friend, you). Participants then had to judge whether shape–label pairs were as originally presented (square–stranger, triangle–friend, circle–you) or whether they were re-paired (square–friend, triangle–you, circle–stranger). After forming these associations, participants are typically faster at judging whether shape–label pairs match for the self and familiar other stimuli compared with shape–label pairs for unfamiliar others; this effect is most pronounced for self-related stimuli (Sui et al., 2012). In this study, we assessed the idea that the enhanced perceptual matching and enhanced memory effects found for self-related stimuli specifically reflect stronger binding (e.g., in the perceptual matching task between the neutral geometric shape and the personal label). The binding account makes two specific predictions. One is that there should be enhanced matching performance for self-related stimuli. As we have noted, this has been found previously (Sui et al., 2012). However, there is a flipside to the effects of binding, which is when a new association has to be formed to a shape previously linked to the self. In this case, the prior binding of the shape to the self may make it difficult to re-form a new association of the shape to a different personal label (e.g., to a friend or to a stranger): There should be a negative effect of strong binding on forming a new association. We evaluated this here. In two experiments, participants first matched geometric shapes against labels corresponding to the self, a friend, or a stranger. If self-reference enhances binding, then we would expect that there would be faster and more accurate responses to new associations formed to self-associated stimuli (compared with stimuli associated with other people). This should facilitate the matching of the new self-associated stimuli (see Sui et al., 2012). The converse of this, however, should occur when self-associations have to be discarded. This is the case when a self-associated shape from the first association is presented as a mismatch stimulus after the shape–label assignments have been switched. Here, the presence of strong “associative glue” may disrupt performance, as it should be difficult to retract the earlier matching response to the self-associated shape.

These proposals were assessed in two experiments. In Experiment 1, after the initial matching phase, the self-associated shape was reassigned to the stranger label. We found that match–mismatch responses were slowed to the former self-associated shapes. However, such a result may stem from re-pairing a familiar association (e.g., circle–you, square–friend) to form a new association to a label for an unfamiliar person on match trials (circle–stranger) and a familiar person on mismatch trials (e.g., circle–friend). In Experiment 2, the self-associated shape was re-paired with the label for a friend, so that switching always created familiar referents on match trials (circle–you and square–friend to circle–friend) and the former self shape was paired with an unfamiliar referent on mismatch trials (circle–stranger). This rules out the possibility that slow performance with shapes formerly associated with the self was due to switching between familiar and unfamiliar referents. In addition, by replicating the results across two experiments, we demonstrate the stability of the effects and provide a more powerful test of the relations between the benefits and costs of self-binding (on forming new associations and switching associations), correlating one effect against the other across participants. Both experiments involved two parts. In Part 1, participants formed associations between personal labels (self, friend, and stranger) and individual shapes (square, triangle, and circle). In Part 2 (swap trials), the shapes and labels were re-paired and participants carried out a sequential matching task in which they saw a shape followed by a label and they had to respond on the basis of whether the shape–label pair corresponded to the new assignments (on match trials, they did; on mismatch trials, shapes and labels were re-paired). We evaluated self-related associative matching both on the initial learning phase (Part 1) and on trials in which participants needed to switch associations (Part 2). Is matching facilitated to self-related stimuli in the initial association block (Part 1)? Conversely, are there costs involved in breaking the old association of a shape to the self when the self-related shape is subsequently linked to another label (in Part 2)?

## Experiment 1: Reassignment 1 (Self to Stranger, Friend to Self, Stranger to Friend)

### Method

#### Participants

Twenty-one college students took part (three men; the age range was 19–27 years, *M* = 22 years, *SD* = 2.82). The sample size was determined by the prior studies to achieve a reasonable effect size (e.g., Sui et al., 2012). All participants were right-handed and had normal or corrected-to-normal vision. Informed consent was obtained based on procedures approved by a local ethics committee.

#### Stimuli and tasks

There were two phases in this experiment. Participants first had to learn shape–personal label associations (in Part 1) followed by a second association matching task in which the shape–label assignments were switched (in Part 2). There were three types of geometric shape (triangle, square, and circle). The shapes subtended 3.6° × 3.6° of visual angle in width and height, respectively, and a central fixation cross subtended 0.8° × 0.8° of visual angle. The labels subtended 1.3–2.6° × 1.3° of visual angle in width and height, respectively. In the first phase, shapes were randomly assigned to three personal labels (friend, you, stranger). The order of assignment was counterbalanced across participants. For instance, participants could be told that a triangle represented their best, gender-matched friend (named by participants before the experiment), a square represented themselves, and a circle was associated with a gender-matched stranger (which was chosen by participants from a list of common names). After the associative instruction, participants had to choose which of the three labels matched the shape in a display. For this task, in which the shape appeared above fixation and the three labels fell below, participants had to choose which label matched the shape by pressing one of three keys. The center of shape and labels fell 3.2° away from fixation and the labels were randomly assigned to set locations for each participant (left, central, right). The task was terminated after six consecutive correct responses to each shape were made.^1^ After the association task, the shape–label assignments were switched. Participants were verbally instructed that the self-associated shape was now assigned to the stranger, the friend-associated shape was now assigned to the self, and the stranger-associated shape was now assigned to the friend. They then received trials in which a centrally presented shape was followed by a central label and the task was to judge whether the shape–label pair matched the switched relations.

The experiment was run on a personal computer with a 21-in. monitor (1,024 × 768 pixels, 100 Hz) using E-Prime software (Version 2.0).

#### Procedure

The matching task (with shape–label assignments switched) was preceded by the association task. In the association task (Part 1), a block started with a central fixation cross for 2,000 ms. On each trial, a shape was paired with three labels displayed for 1,000 ms, followed by a blank screen with 2,000 ms as maximal duration. Participants had to make a response during this 3,000-ms time window using one of three response keys on a keyboard. The display was terminated by a response (*B*, *N*, and *M* keys for the left, central, and right stimuli, respectively). Feedback was given for 500 ms after the response. The experiment began after six practice trials. The task was terminated by six consecutively correct responses to each association. Participants took on average 4.10 min to get to the criterion in the association task in Part 1, and the variability across participants was 0.69∼8.20 min.

In the matching task (Part 2), participants were instructed to reassign the shape–label relations: (a) the self-associated shape was linked to the stranger label, (b) the friend-associated shape was assigned to the you label, and (c) the stranger-associated shape was assigned to the friend label. After this instruction, participants undertook a shape–label matching task. The central fixation appeared for 500 ms. A shape was displayed for 100 ms, followed by a blank screen for 200 ms. After this, a label was displayed for 100 ms and then the blank screen was presented again for 1,000 ms, during which time participants had to judge whether the shape and label matched using one of two response keys (*Z* or *M* keys; keys assigned for match and mismatch responses were counterbalanced over participants). Feedback (“correct,” “incorrect,” or “slow”) was given for 500 ms. There were nine experimental conditions (3 shapes × 3 labels). There were eight blocks of 81 trials in total, including nine practice trials. There were thus 72 trials per condition.

We also ran a control experiment with an independent set of participants who received the instructions for Part 2 only and who then judged whether shape–label pairings matched the instructions. This control condition provided a baseline to assess whether there was a cost on mismatch trials to shapes that would have been linked to the self on match trials (in the full Experiment 1), without any of the shapes previously having been associated to the self (as in Part 1 here). In the control experiment, there were 22 college students (13 men; the age range was 19–32 years, *M* = 23.78 years, *SD* = 4.11). The method was identical to Part 2 (the switching task) in the experimental condition.

#### Data analyses

We report the error rates and reaction times (RTs).

First, we conducted repeated measures analyses of variance (ANOVAs) with one factor—association (self, friend, or stranger)—to examine the effect of self and familiar other associations on identifying shape–label pairings (Part 1).

Second, we measured the self- and friend biases compared with the stranger association in the matching task (after switching, in Part 2). We first assessed whether there was a self-bias in forming the new associations on shape–label match trials (Part 2). We then evaluated any difficulties in re-pairing the old self-shape with a new label on mismatch trials (Part 2). For this latter analysis, we removed the exact pairings of the shapes and labels used in Part 1 so that mismatch RTs were not contaminated by including the pairs that were originally associated.

All multiple comparisons were corrected by multiple Holm–Bonferroni corrections for α = .05 (Holm, 1979).

### Results

#### Self-bias in the association task (Part 1)

**Figure 1 fig1:**
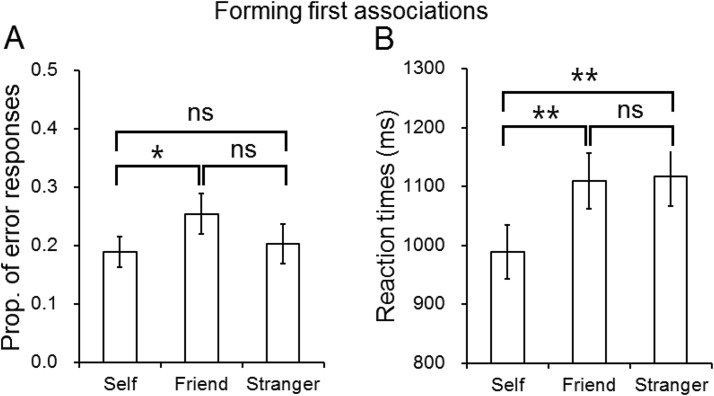
The performance in the error rates and reaction times (ms) in corrected trials as a function of association (self, friend, or stranger) in the association task in Experiment 1. The error bars represent 1 standard deviation. ns = not significant. * *p* < .05. ** *p* < .01.

A repeated measures ANOVA was conducted on error rates with one within-subjects factor—association (self, friend, or stranger). This showed a significant effect of association, *F*(2, 38) = 3.35, *p* < .05, η^2^ = .15; fewer errors were made in forming self-than friend associations, *t*(19) = −2.75, *p* = .013. There was a trend for fewer errors to stranger than friend associations, but the effect was not significant, *t*(19) = 1.86, *p* = .08. There was no difference in errors between the self- and stranger associations, *t*(19) = −0.49, *p* = .63 (see Figure 1A).[Fig-anchor fig1]

A similar analysis on RTs demonstrated a significant effect of association, *F*(2, 38) = 10.24, *p* < .001, η^2^ = .35; there were faster responses to self-associations than friend associations, *t*(19) = −3.60, *p* = .002, and also stranger associations, *t*(19) = −4.12, *p* = .001; there was no difference between the friend and stranger associations, *t*(19) = −0.25, *p* = .80 (see Figure 1B).

The data indicated a reliable advantage for matching self-associations compared with friend and stranger associations.

#### Self-bias following switching (Part 2)

##### Forming reassigned associations (match trials)

**Figure 2 fig2:**
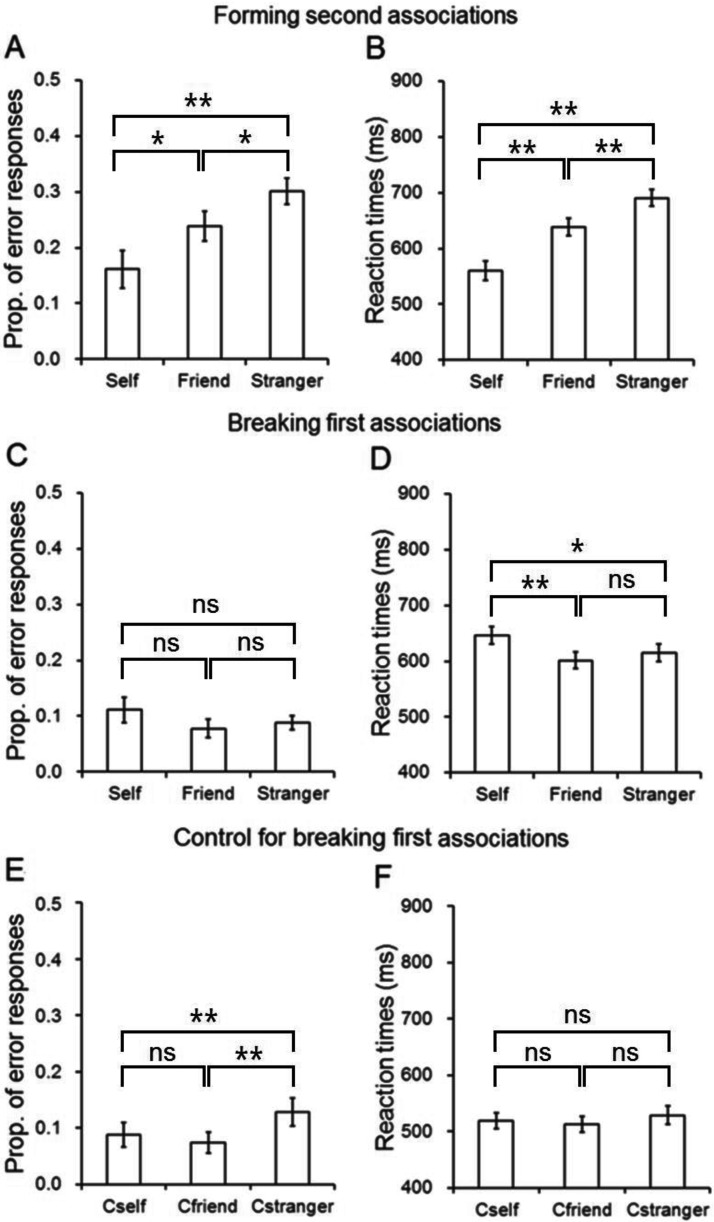
The performance in the switch task in Experiment 1. (A, B) Forming second associations after shape–label re-pairing in the switch task, data in label-based match trials as a function of association (self, friend, or stranger) in the error rates and reaction times (ms) in corrected trials. (C, D) Breaking first associations formed in the association task, data based on the former shapes on mismatch trials as a function of association (self, friend, or stranger) in the error rates and reaction times (ms) in corrected trials. (E, F) Performance in the control task was extracted to match those in breaking first associations as a function of association (self, friend, or stranger) in the error rates and reaction times (ms) in corrected trials. Cself, Cfriend, and Cstranger refer to the self, friend, and stranger associations in the control task respectively. The error bars represent 1 standard deviation. ns = not significant. * *p* < .05. ** *p* < .01.

**Figure 3 fig3:**
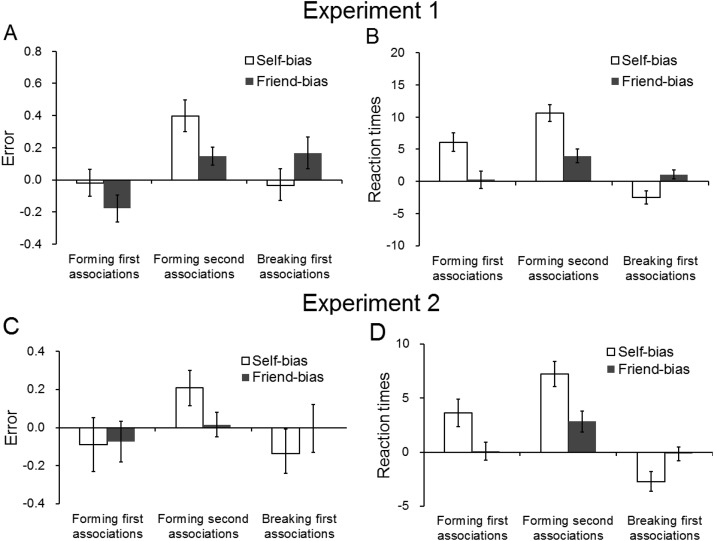
The cost of switching in self- and friend bias in forming first association, breaking first association, and forming second associations in the error rate (A, C) and reaction times (B, D) in Experiments 1 and 2. The data on reaction times were indexed by the differential scores between the stranger and self (or friend) divided by the sum of the two conditions and then multiplying by 100. The data on errors were indexed by the differential scores between the stranger and self (or friend) divided by the sum of the two conditions. The error bars represent 1 standard deviation.

The analysis of the error rate on match trials showed a significant effect of association, *F*(2, 38) = 7.65, *p* = .002, η^2^ = .29 (see Figure 2A); there were fewer errors when matching self-related stimuli than stranger-related stimuli, *t*(19) = −3.31, *p* = .004, marginally fewer errors when matching friend relative to stranger associations, *t*(19) = −2.41, *p* = .026, and marginally fewer errors when matching self-relative to friend associations, *t*(19) = −2.07, *p* = .05 (see Figure 3A).[Fig-anchor fig2][Fig-anchor fig3]

The analysis of RTs also showed a significant effect of association, *F*(2, 38) = 43.29, *p* < .001, η^2^ = .70 (see Figure 2B); there were faster responses in matching self-associated stimuli than friend-associated items, *t*(19) = −6.00, *p* < .001, and stranger-associated stimuli, *t*(19) = −8.54, *p* < .001; there were also faster responses to friend than stranger associations, *t*(19) = −3.73, *p* = .001 (see Figure 3B).

##### Breaking first associations (mismatch trials)

The trials were sorted according to whether the shape was associated with the self, the friend, or the stranger label in Part 1 (the association task). There was no effect of shape on errors on mismatch trials, *F*(2, 38) = 1.66, *p* = .20, η^2^ = .08 (see Figures 2C and 3A). However, the analysis on RTs did demonstrate a significant effect of shape, *F*(2, 38) = 7.34, *p* = .002, η^2^ = .28 (see Figure 2D). There were slower responses to mismatch trials with the self-associated shape compared with those with the friend-, *t*(19) = 3.23, *p* = .004, and the stranger-associated shape, *t*(19) = 2.38, *p* = .028; the latter two conditions did not differ, *t*(19) = −1.67, *p* = .11 (see Figure 3B).

The results demonstrate that it was hard to make a mismatching response to a shape when it was previously associated with and matched to a self label compared with shapes that were previously associated with and matched to other labels (friend and stranger).

#### Comparisons with the control task

The data for the three shapes in the mismatching conditions of the control experiment were extracted. A repeated measures ANOVA was conducted on the error results with one between-subjects factor (task: experimental vs. control task) and one within-subjects factor (association: self, friend, or stranger). There was no significant main effect of task, *F*(1, 40) = 0.03, *p* = .87, η^2^ = .00. The effect of association was significant, *F*(2, 80) = 4.20, *p* = .02, η^2^ = .10; there were fewer errors for friend relative to stranger associations (*p* = .007), but there were no differences between the self- and friend associations (*p* = .18) nor between the self- and stranger associations (*p* = 1.0). There was also a significant interaction between task and association, *F*(2, 80) = 4.08, *p* = .02, η^2^ = .09. For the control task, the error analysis showed a significant effect of association, *F*(2, 42) = 1.66, *p* = .20, η^2^ = .08 (see Figure 2E); there were fewer error responses to the self, *t*(21) = −2.97, *p* = .007, and friend, *t*(21) = −3.89, *p* = .001, shapes on mismatching trials compared with the stranger association, but there was no difference between the self- and friend associations, *t*(21) = 1.04, *p* = .31. We also conducted comparisons between the switch task and control task. To rule out task difficulty, we conducted pairwise *t* tests on the relative scores (self-bias = stranger—self; friend bias = stranger—friend) between two tasks. The analyses revealed a significant difference in self-biases between the two tasks, *t*(40) = −2.62, *p* = .01, indicating fewer errors in the control task relative to the switch task. Similarly, there was a significant difference in friend biases between the two tasks, *t*(40) = −2.24, *p* = .03, indicating fewer errors in the control task than in the switch task. In contrast, the analysis on RTs failed to show a significant effect of association in the control task, *F*(2, 42) = 2.75, *p* = .08, η^2^ = .12 (see Figure 2F).

The data demonstrated different patterns on mismatch trials between the experimental task (after switching shape assignments) and the control task (confirmed by the Task × Association interaction), with the self-disadvantage emerging only for the switch experiment. The results indicate that the difficulty in responding to a self-associated shape on mismatch trials reflected the switching cost following the first association task rather than being a simple product of the matching task (e.g., a bias on mismatch trials against the shape paired with the self on match trials).

In summary, the analyses showed benefits for self-related stimuli when matches were made to the initial shape–label associations (in Part 1). Following switching of the shape–label pairs (Part 2), match responses to new associations to the self were still advantaged. However, there was now a cost on mismatch trials for shapes that were formerly related to the self. This result was not found in a control experiment without earlier switching of the shape–label assignments.

## Experiment 2: Reassignment 2 (Self to Friend, Friend to Stranger, Stranger to Self)

### Method

There were 20 college students (12 men; the age range was 18–30 years, *M* = 23.70 years, *SD* = 3.51). The sample size was determined by both Experiment 1 and prior studies to achieve a reasonable effect size (e.g., Sui et al., 2012). Participants were right-handed and had normal or corrected-to-normal vision. Informed consent was obtained based on procedures approved by a local ethics committee.

The method in Experiment 2 was identical to that in Experiment 1, except that in the switch task, the initial self-associated shape (in Part 1) was reassigned to the friend label (in Part 2), the friend-associated shape in Part 1 was reassigned to the stranger label in Part 2, and the stranger-associated shape in Part 1 to the self label on match trials in Part 2. On mismatch trials, the initial self-associated shape was paired with either the self or stranger label, the initial friend-associated shape was paired with the self or friend label, and the initial stranger-associated shape was paired with the friend or stranger label. As in Experiment 1, the data analysis on mismatch trials was based on only the newly paired shape–label associations (extracting out data for trials in which the shape and label matched the original assignment in Part 1). Participants took about average 2.38 min to get to the criterion of the association task in Part 1, and the variability across participants was 0.90∼5.80 min.

### Results

#### Self-bias in the association task (Part 1)

**Figure 4 fig4:**
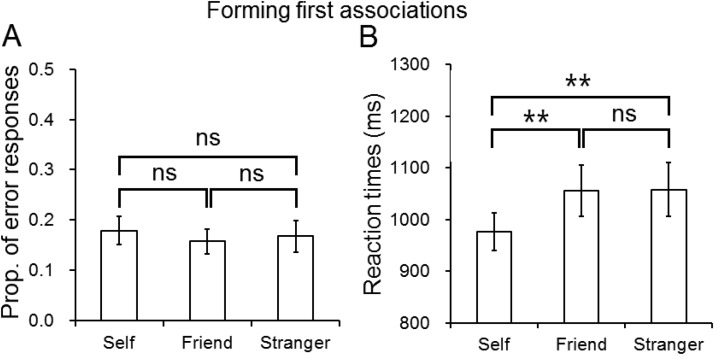
The performance in the error rates and reaction times (ms) in corrected trials as a function of association (self, friend, or stranger) in the association task in Experiment 2. The error bars represent 1 standard deviation. ns = not significant. ** *p* < .01.

A repeated measures ANOVA on errors failed to show a significant effect of association, *F*(2, 38) = 0.02, *p* = .98, η^2^ = .001 (see Figure 4A). The analysis on RTs showed a significant effect of association, *F*(2, 38) = 8.10, *p* = .001, η^2^ = .30 (see Figure 4B); there were faster responses to self-than friend, *t*(19) = −3.04, *p* = .007, and stranger associations, *t*(19) = −3.16, *p* = .005, but there was no difference between the friend and stranger associations, *t*(19) = 0.07, *p* = .94. The data indicated a reliable advantage for matching self-associations than for matching friend and stranger associations.[Fig-anchor fig4]

#### Self-bias in the switching task (Part 2)

##### Forming reassigned associations (match trials)

**Figure 5 fig5:**
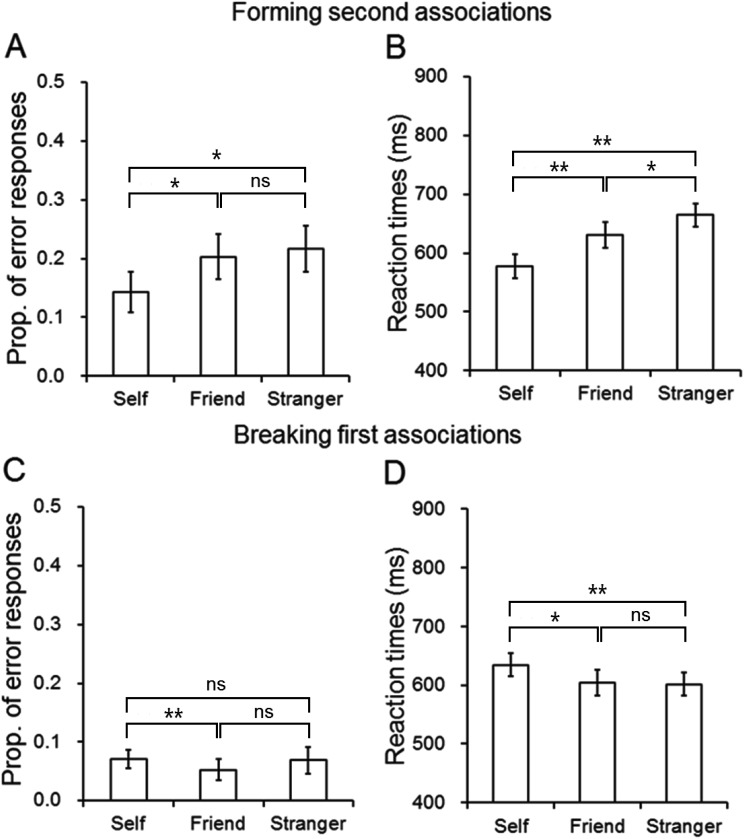
The performance in the switch task in Experiment 2. (A, B) Forming second associations after shape–label re-pairing in the switch task, data in label-based match trials as a function of association (self, friend, or stranger) in the error rates and reaction times (ms) in corrected trials (C, D). Breaking first associations formed in the association task, data based on the former shapes on mismatch trials as a function of association (self, friend, or stranger) in the error rates and reaction times (ms) in corrected trials. The error bars represent 1 standard deviation. ns = not significant. * *p* < .05. ** *p* < .01.

The analysis on errors showed a significant effect of association, *F*(2, 38) = 3.93, *p* = .03, η^2^ = .17 (see Figure 5A); there were marginally fewer errors in responding to second self-associations than in responding to stranger associations, *t*(19) = −2.39, *p* = .027, and friend associations, *t*(19) = −2.22, *p* = .039; there was no difference in errors between friend and stranger associations, *t*(19) = −0.49, *p* = .63 (see Figure 3C).[Fig-anchor fig5]

For the RT data, there was a significant effect of association, *F*(2, 38) = 17.35, *p* < .001, η^2^ = .48 (see Figure 5B); there were faster responses to the new self-associations than the new friend, *t*(19) = −3.37, *p* = .003, and stranger associations, *t*(19) = −5.56, *p* < .001, and faster responses to friend than stranger associations, *t*(19) = −2.50, *p* = .022 (see Figure 3D).

In line with the results in the association task (Part 1), the analyses in the switch task showed that, although shapes and labels were re-paired, there was a robust self-advantage in forming new associations compared with the friend and stranger conditions.

##### Breaking first associations (mismatch trials)

There was an effect of association on errors on mismatch trials, *F*(2, 38) = 3.96, *p* = .027, η^2^ = .17 (see Figure 5C); there were more errors to former self shapes than to former friend shapes, *t*(19) = 3.22, *p* = .004. There were trends for more errors to former self shapes than stranger shapes and for more errors for former stranger shapes than friend shapes, but these effects were not reliable, *t*(19) = 1.14, *p* = .27, and *t*(19) = −1.46, *p* = .16 (see Figure 3C).

The analysis on RTs demonstrated a significant effect of association, *F*(2, 38) = 6.17, *p* = .005, η^2^ = .25 (see Figure 5D); there were slower responses to shapes formerly associated with the self than the friend, *t*(19) = 2.60, *p* = .018, and stranger, *t*(19) = 2.88, *p* = .010. There were no differences in responding to stimuli formerly associated with the friend and stranger, *t*(19) = 0.57, *p* = .57 (see Figure 3D).

The results confirm those found in Experiment 1. In the initial association phase, participants were better at matching self-associated stimuli than stimuli associated to friend and stranger stimuli (Part 1). After switching the shapes and labels, there was also an advantage for match responses to new associations to self labels compared with match responses to new associations to friend and stranger labels (Part 2). In contrast, mismatch responses were disrupted for shapes that were previously associated to the self (Part 2). The pattern of these results is presented in Figure 3.

#### Correlation results

We evaluated the relations between forming the new association on match trials in Part 2 of each experiment and breaking the old association on mismatch trials in Part 2. If self-association enhances binding, then participants who find it easier to bind new stimuli to the self (who show a larger self-advantage on match trials) will also find it more difficult to break the old self-related association, when the shape formerly associated to the self is reassigned a new pairing (showing a larger cost on mismatch trials). To test this, we correlated the self-advantage on match trials in Part 2 with the self-cost on mismatch trials.

**Figure 6 fig6:**
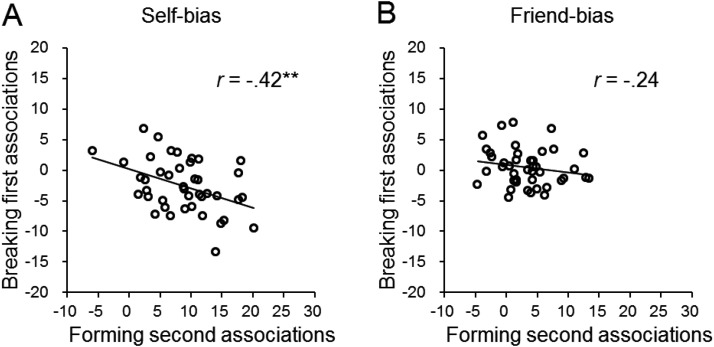
The correlations in reaction times between forming second associations and breaking first associations in self-bias (A) and friend bias (B) across the two experiments. ** *p* < .01.

To maximize power, we merged data in the two experiments, first normalizing RTs to provide indices of the self-/friend bias—the differential scores between the stranger and self (or friend) divided by the sum of the two conditions and then multiplying 100. This means that a self-advantage on matching trials in Part 2 shows as a positive value, whereas a self-cost on mismatching trials shows as a negative value. Pearson correlation analyses showed a significant negative correlation between the self-advantage when forming a new association on match trials and the disadvantage on making mismatch responses to former self-related stimuli, *r*(40) = −.42, *p* = .008 (see Figure 6A). This shows that, the greater the self-bias on forming a new association on match trials, the greater the cost of breaking the initial self–shape association on mismatch trials. There was a similar trend for a relationship between the advantage for forming a new association linked to the friend label on match trials and the cost on mismatch trials to the former friend-related stimulus, but it failed to reach significance, *r*(40) = −.24, *p* = .15 (see Figure 6B). However, there was no significant difference in the magnitudes of the self and friend correlations using Fisher’s *r*-to-*z* transformation, *p* = .46. The analyses on the errors did not reveal any significant correlations, *r*(40) < .51, *p* > .51.[Fig-anchor fig6]

## Discussion

Similar to previous studies on simple shape–label associative learning, we have shown that participants are biased in their responses to self-related stimuli compared with stimuli related to other people (here, a best friend or a stranger; Sui et al., 2012). This self-advantage occurred when participants had to match one of three labels to a shape (Part 1) and when they sequentially matched a shape to a label (Part 2). Critically, we examined the effects of re-pairing shapes and label after the initial associations had been switched (Part 2). Here, we found three main results: (1) New associations to a self label were responded to faster than new associations to the other labels (friend and stranger; match trials in Experiments 1 and 2), (2) it was more difficult to make mismatch responses to the self shape when it was paired with a new label (mismatch trials in Experiments 1 and 2), and (3) the advantage for self-related shapes on forming new match associations was correlated with the cost to rejecting former self-associated shapes on mismatch trials.

These results can be accounted for by the hypothesis that associating stimuli with the self benefits from a stronger memorial glue than associating stimuli with representations for other people. This means that self-associations are in general stronger than associations to other people and support enhanced RTs to match stimuli conforming to new self-association. This was demonstrated here in Part 1 of each experiment. These data match many other results in the literature demonstrating that self-association enhances memory, including the binding of the memory to its source (Cunningham et al., 2008, 2011; Rogers et al., 1977; Sui & Humphreys, 2013). It also means that new associations of shapes formerly paired with other stimuli (friend, stranger) can be rapidly assimilated with a self label when new associations must be formed. In this sense, it appears to be relatively easy to expand a self-representation by increasing the number of external stimuli that are linked to it. We suggest that the enhanced binding process means that we can rapidly acquire knowledge in relation to ourselves, and that this process is facilitated relative to when knowledge is acquired about other people. It is interesting to speculate whether this rapid acquisition of associations to the self is helpful for developing a self-representation.

There is also evidence for self-association improving perceptual matching (Sui et al., 2012) and facilitating perceptual integration (measured using redundancy gains; Sui & Humphreys, 2015; Sui, Yankouskaya, & Humphreys, 2015). Sui et al. (2015) evaluated effects of redundancy gains with stimuli related to the self or to other people. Redundancy gains occur when responses are facilitated when two targets are presented relative to when participants respond to a single target (Mordkoff & Danek, 2011; Yankouskaya, Booth, & Humphreys, 2012). In Sui et al. (2015), participants first formed shape–label associations and then they had to make identification responses to either single or two-shape exemplars (you or friend?). Redundancy gains were larger for pairs of self-related shapes compared with shapes related to a friend association. Moreover, for self shapes only, these redundancy gains violated assumptions based on the independent processing of the shapes (cf. Miller, 1982) and self-related shapes were processed with “supercapacity,” whereas friend-related shapes showed mutual interference (Townsend & Eidels, 2011). These results suggest that self-related stimuli benefit from enhanced perceptual integration in their processing, so that responses to a “whole” two-item pair are greater than responses to a single item. These latter results are consistent with self-association also providing a form of “perceptual glue” in addition to the evidence for increased memorial glue that we have presented here. The converging evidence shows that self-reference has a broad influence on binding information.

However, the other side of forming an association that is strongly bound to the self is that it becomes difficult to break that association when a formerly associated shape must be linked to another person: It was difficult for participants to make mismatch responses to shapes formerly associated with the self.

The factors that lead to these stronger binding effects for self-related items remain unclear. For example, it has been argued that self-biases may reflect underlying factors such as the reward value (Northoff & Hayes, 2011) or the emotional value linked to self-related items (Ma & Han, 2010). For example, there may be enhanced processing of self-related stimuli because they have intrinsic reward value and/or because they have positive emotional valence. There is evidence that brain structures such as the hippocampus are critically involved in binding features in memory and that hippocampal activity is modulated by reward. Indeed, brain imaging indicates that the hippocampal structures that discriminate reward are also involved in binding associations in memory (Wolosin, Zeithamova, & Preston, 2012, 2013). Similarly, there is evidence that positive emotion can enhance integrative associative memory (Murray & Kensinger, 2013, 2014). Thus, both reward and emotion are candidate “drivers” for the binding effects we observe in relation to the self. On the other hand, Sui et al. (2015) demonstrated that perceptual integration from reward-related stimuli was not as pervasive as the integration found with self-related items and suggested that perceptual binding related to the self was distinct and not modulated by reward. Whether the same holds for binding in memory is an empirical question. Stolte, Humphreys, Yankouskaya, and Sui (2015) have also shown that self-biases and emotion-related biases are not correlated, although this would be expected if they reflect common underlying processes. Clearly, an important question for future work will be to tie down what factors are critical for the enhancement of binding by self-reference. It is nevertheless interesting to note that we did not find strong evidence here for costs of binding for shapes formerly linked to friend-related stimuli, although friend associations did benefit in matching (at least for Experiment 2). Also, although there were trends for a correlation between the matching advantage for friend-related stimuli and the disadvantage for mismatching stimuli formerly related to the friend, this was not reliable.^2^ Although we should be cautious in our interpretation, the data indicate that differential effects of familiarity may help new learning but do not necessarily impair the reassignment of old relations. The difficulty in reassigning self-related associations may reflect a factor other than familiarity.

We conclude that self-association can either enhance or disrupt processing, depending on whether new associations are assessed or whether old associations have to be discarded. The results fit with the idea that self-association leads to stronger binding in memory, a property that may facilitate the development of our self-representations.

## Footnotes

1 This procedure differs from the perceptual matching task reported in Sui et al. (2012) and in Part 2 of this study. This was because, in Part 1, we were interested primarily in assessing whether there was a self-advantage on matching trials. The current procedure maximized the number of matching trials as no mismatch trials were presented.

2 Although we note that the difference in the magnitudes of the correlations to self- and friend-related stimuli did not differ.
